# Supplementary material for: Nuclease‐Resistant L‐DNA Tension Probes Enable Long‐Term Force Mapping of Single Cells and Cell Consortia
Source: Angew Chem Int Ed Engl. 2024 Oct 25;63(50):e202413983. doi: 10.1002/anie.202413983 (PMC11610648; doi:10.1002/anie.202413983)
Supplement: Supplementary file 1 — Supporting Information [file ANIE-63-e202413983-s001.pdf]

Angewandte Chemie
Eine Zeitschrift der Gesellschaft Deutscher Chemiker

Supporting Information

Nuclease-Resistant L-DNA Tension Probes Enable Long-Term Force Mapping of Single Cells and Cell Consortia

*S. Sethi, T. Xu, A. Sarkar, C. Drees, C. Jacob, A. Walther**

Supplementary Information

Nuclease-Resistant L-DNA Tension Probes Enable Long-Term Force Mapping of Single Cells and Cell Consortia

Soumya Sethi[a], Tao Xu[a], Aritra Sarkar[a], Christoph Drees[a], Claire Jacob[b], and Andreas Walther*[a]

[a] Dr. S. Sethi, T. Xu, Dr. A. Sarkar, Dr. C. Drees and Prof. A. Walther
Life-like Materials and Systems, Department of Chemistry
University of Mainz
Duesbergweg 10–14, 55128 Mainz (Germany)
E-mail: andreas.walther@uni-mainz.de

[b] Prof. C. Jacob
Department of Biology
University of Mainz
Hanns-Dieter-Hüsch-Weg 15, 55128 Mainz (Germany)

Contents

Nuclease-Resistant L-DNA Tension Probes Enable Long-Term Force Mapping of Single Cells and Cell Consortia 1

Experimental Materials and Methods 3

1. Materials 3
2. General Characterization Methods and Instruments 3
   - 2.1 TIRF and Brightfield Microscopy 3
   - 2.2 DNA Concentrations 3
   - 2.3 Statistical Analysis 3
   - 2.4 MALDI-ToF MS 3
   - 2.5 DNA Degradation Assay 3
3. Methods 3
   - 3.1 Surface Preparation 3
   - 3.2 DNA Hybridization 4
   - 3.3 DNA Oligonucleotide Coupling to Cyclic RGD and Purification 4
   - 3.4 Cell Culture 4
   - 3.5 Background Subtraction Methods 4
   - 3.6 Signal-to-Noise Ratio Calculations 4
4. Supplementary Tables 5
5. Supplementary Figures 6
6. Supplementary References 14

Experimental Materials and Methods

1. Materials

Cyclo[Arg-Gly-Asp-d-Phe-Lys(PEG-PEG-azide)] (RGD-3759-PI) was purchased from biosynth. Neutravidin (31000) (Thermo-Fisher Scientific), Bovine Serum Albumin biotinylated (29130) (Thermo-Fisher Scientific), 25 mm x 75 mm glass coverslips (10812), and sticky slide 8 well (80828) were purchased from ibidi. All oligonucleotides were purchased from Biomers. All buffers were prepared with nuclease free water.

2. General Characterization Methods and Instruments

2.1 TIRF and Brightfield Microscopy

TIRF and Brightfield Microscopy were performed on a Zeiss Elyra 7 Imaging System equipped with 405 nm, 488 nm, 561 nm, and 642 nm excitation lasers using alpha Plan-Apochromat 63x, N.A. 1.46.oil immersion, TIRF objective, pco.edge 4.2 CLHS water-cooled sCMOS cameras. Microscopy experiments were typically conducted using the following parameters: 642 nm laser: 0.5% output (~0.6 mW). 405/488/561/642 quadband dichroic mirror and 655-720 nm bandpass emission filter. Exposure time: 300 ms. TIRF angle: 66° (n_{oil}: 1.518). Live cell imaging was conducted at 37 °C in humidified air with 5% CO_2 for NIH-3T3 cells and 10% CO_2 for A-10 cells. All images were contrasted equally.

2.2 DNA Concentrations

DNA concentrations were determined using a DeNovix-S-06873 (DeNovix OS 0.8.1 v4.1.5) spectrophotometer with a standard value of 33 µg/OD_{260}.

2.3 Statistical Analysis

p values were calculated by performing a t-Test (Two-Sample Assuming Equal Variances) in Microsoft Excel using a built-in data analysis tool pack.

2.4 MALDI-ToF MS

Matrix-assisted laser-desorption-ionization time of flight mass spectrometry (MALDI-ToF MS) measurements were performed using matrix consisting of 3-hydroxy picolinic acid and diammonium citrate. The measurements are conducted on an autoflex maX MALDI-toF-MS from Bruker.

2.5 DNA Degradation Assay

DNA degradation assay was performed on a TECAN (SPARK CONTROL v3.1) microplate reader using Corning® 384-Well black polystyrene plate with non-binding surface. Excitation and emission wavelengths for Atto647N are 620 nm and 679 nm respectively.

3. Methods

3.1 Surface Preparation

Surface preparation method was adapted from previously published protocols.[1] Briefly, the glass coverslips (25 x 75 mm) were adhered to the sticky slide 8 well slides. Wells were coated with BSA biotin (100 µg/mL) in nuclease free water overnight at room temperature. Wells were rinsed 3 times with nuclease-free water and incubated with 100 µg/mL of neutravidin for 30 minutes at room temperature. Wells were rinsed once more and incubated at room temperature with 200 µL of 100 nM DNA probes for 1 hour. After washing with nuclease free water, cell culture media was added to the well followed by the addition of cells.

3.2 DNA Hybridization

DNA oligonucleotides and DNA hairpins were hybridized at 10 µM in 200 µL PCR tubes and subsequently diluted to 100 nM. DNA oligonucleotides were heated to 95 °C and then cooled at a rate of 1.3 °C/min to 25 °C.

3.3 DNA Oligonucleotide Coupling to Cyclic RGD and Purification

To conjugate cRGDfk to DNA, we used azide/DBCO click chemistry. The DNA was modified with DBCO and the cRGDfk peptide with azide. The reaction was performed in a molar ratio of DNA-DBCO (1 mM)/cRGDfk-N_3 (3 mM) = 1:3 in PBS at 37 °C at 650 rpm for overnight. Excess cRGDfk-N_3 was removed using a 3 kDa MWCO spin filter. The product was confirmed with HPLC and MALDI-ToF MS (Supplementary Figure S10).

3.4 Cell Culture

NIH-3T3 (ACC 59) and A-10 (ACC 132) cell lines were purchased from Leibniz Institute DSMZ-German Collection of Microorganisms and Cell Cultures GmbH and cultured according to the guidelines. Briefly, NIH-3T3 fibroblasts cells were cultured in DMEM supplemented with 10% fetal bovine serum (*v/v*) and penicillin/streptomycin in an incubator with 5% CO_2. A-10 cells were cultured in DMEM supplemented with 20% fetal bovine serum (*v/v*) and penicillin/streptomycin in an incubator with 10% CO_2.

3.5 Background Subtraction Methods

Background subtraction methods were adapted from previously published protocols.[2] Briefly raw images were exported to FIJI (software) followed by selecting 3 ROIs in the fluorescent channel, next the background intensity was measured and subtracted from the images.

3.6 Signal-to-Noise Ratio Calculations

We carried out the process by first calculating the mean fluorescence intensity per cell for every timeframe, followed by selecting three regions of interest (ROIs) adjacent to the cell, for each image and measuring the mean background intensity.

$$Signal - to - noise\ ratio = \frac{\text{Mean fluorescence intensity signal per cell}}{\text{Background mean fluorescence intensity adjacent to cell}}$$

4. Supplementary Tables

Table S1. DNA sequences for the oligonucleotides with their abbreviations, sequence, and modifications.

Name	Sequence 5'→ 3'	Figure	5'Modification	3'Modification
D-DNA Unzipping Mode	GAG GAG GGC AGC AAA CGG GAA GAG TCT TCC TTT ACG TTT T	Fig. 2c, 4c	ATTO 647N	Biotin
D-DNA Ligand Strand	ACG TAA AGG AAG ACT CTT CCC GTT TGC TGC CCT CCT C	Fig. 2c, 4c	DBCO	BHQ 2
L-DNA Unzipping Mode	GAG GAG GGC AGC AAA CGG GAA GAG TCT TCC TTT ACG TTT T	Fig. 2b, 4b	ATTO 647N	Biotin
L-DNA Ligand Strand	ACG TAA AGG AAG ACT CTT CCC GTT TGC TGC CCT CCT C	Fig. 2b, 4b	DBCO	BHQ 2
D-DNA Anchor strand with BHQ 2	CGCATCTGTGCGGTATTTCACTTT	Fig. 3c	BHQ 2	Biotin
D-DNA RGD strand	TTT GCT GGG CTA CGT GGC GCT CTT	Fig. 3c	DBCO	Cy3B
D-DNA Hairpin Strand	GTG AAA TAC CGC ACA GAT GCG TTT-GCG CGC GCG CGC TTT TGC GCG CGC GCG C-TTT AAG AGC GCC ACG TAG CCC AGC	Fig. 3c	None	None
L-DNA Anchor strand with BHQ 2	CGCATCTGTGCGGTATTTCACTTT	Fig.3b	BHQ 2	Biotin
L-DNA RGD strand	TTT GCT GGG CTA CGT GGC GCT CTT	Fig. 3b	DBCO	Cy3B
L-DNA Hairpin Strand	GTG AAA TAC CGC ACA GAT GCG TTT-GCG CGC GCG CGC TTT TGC GCG CGC GCG C-TTT AAG AGC GCC ACG TAG CCC AGC	Fig.3b	None	None

5. Supplementary Figures

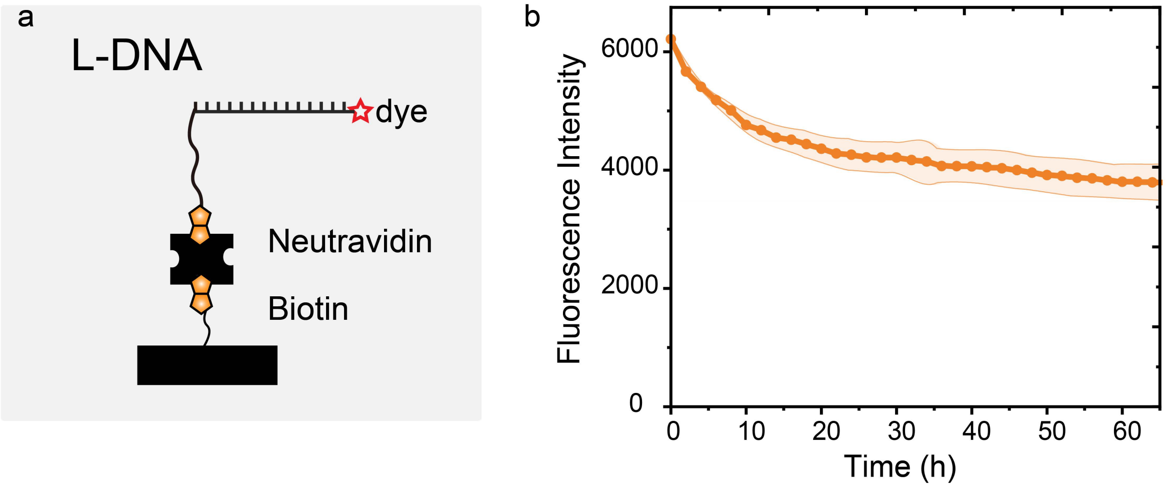

Figure S1. Assessment of stability of L-DNA immobilized on biotin-neutravidin surfaces in cell culture environment at 37 ˚C. (a) Scheme depicting the immobilization of ss-L-DNA. (b) Mean fluorescence intensity (FI) captured using TIRF illumination over a period of 3 days.

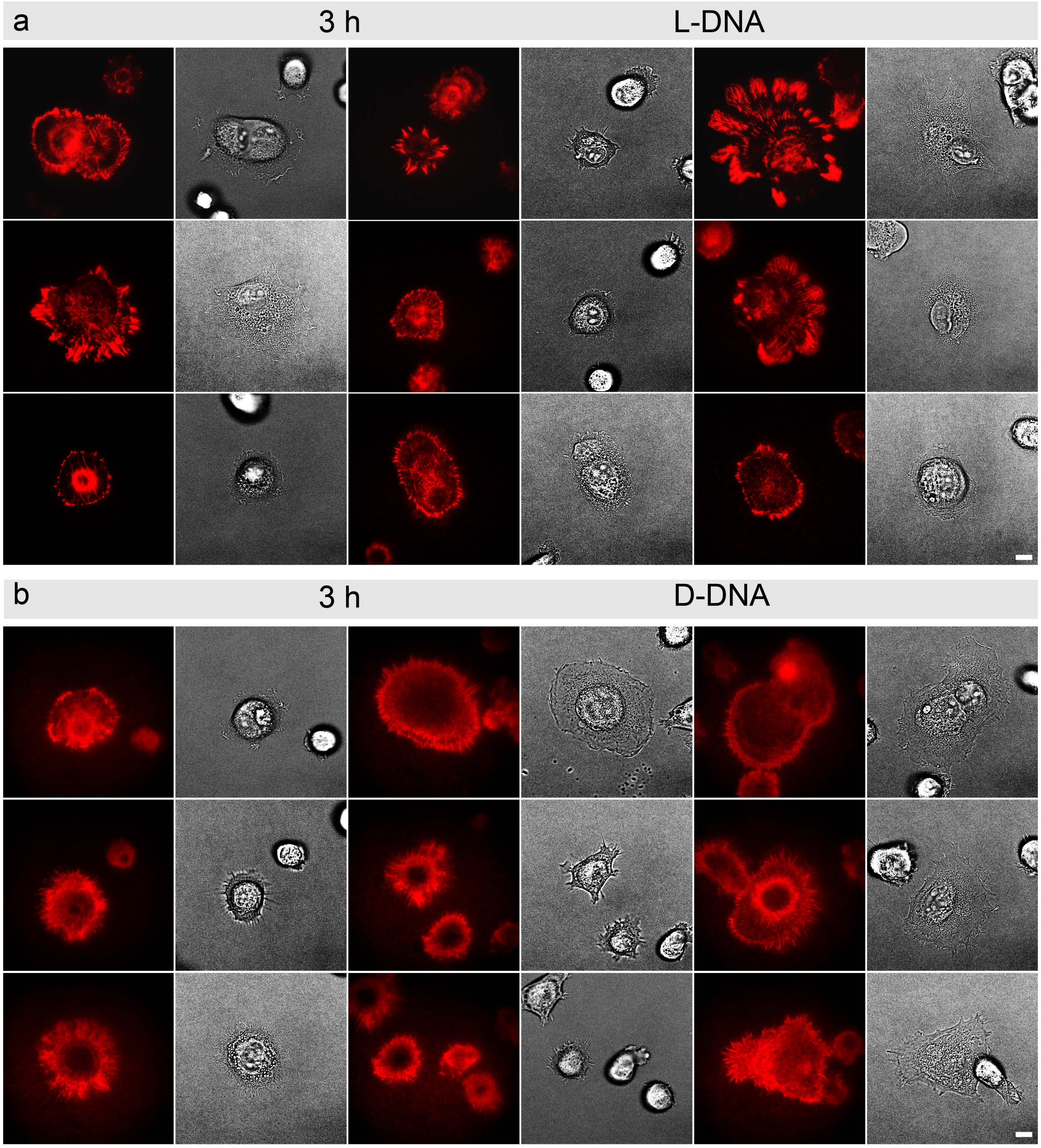

Figure S2. Representative Images of tension signals made by fibroblasts as they adhere, spread, crawl at 3 h time point. (a-b) Brightfield and fluorescence images on (a) L-DNA and (b) D-DNA tension probe surfaces. Scale bars = 10 μm

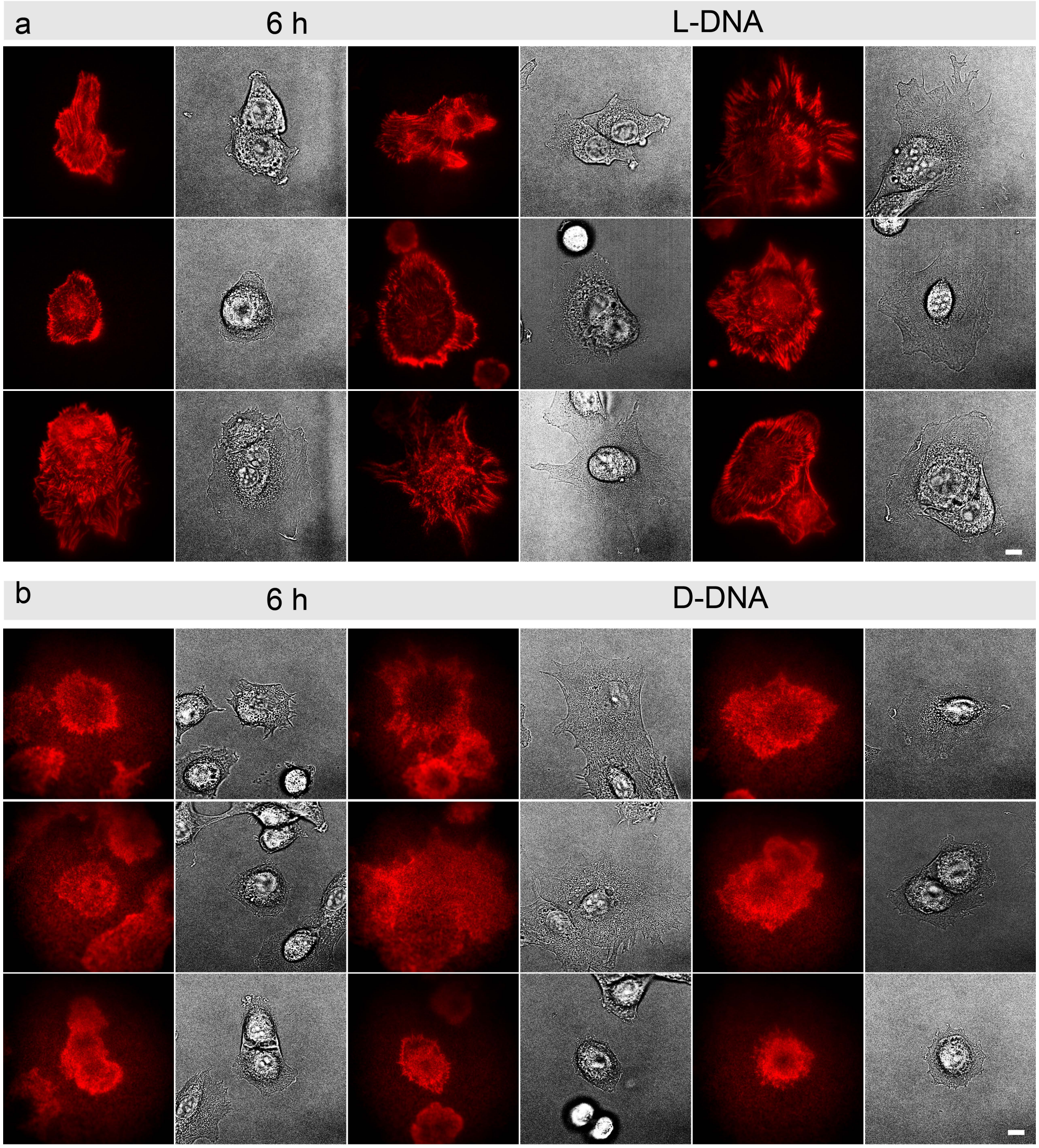

Figure S3. Representative Images of tension signals made by fibroblasts as they adhere, spread, crawl at 6 h time point. (a-b) Brightfield images and fluorescence signals on (a) L-DNA and (b) D-DNA tension probe surfaces. Scale bars = 10 μm

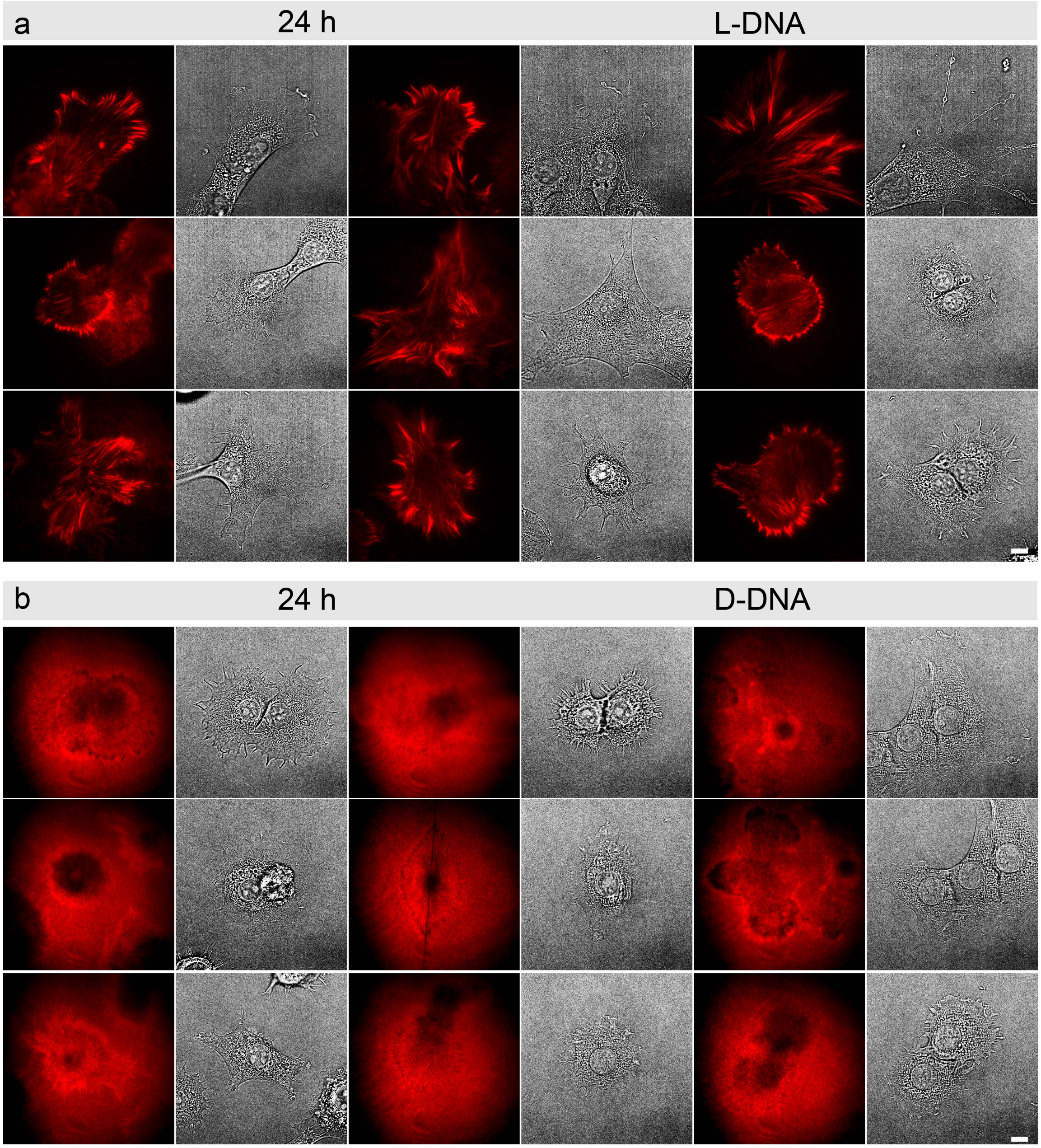

Figure S4. Representative Images of tension signals made by fibroblasts as they adhere, spread, crawl at 24 h time point (a-b) Brightfield images and fluorescence signals on (a) L-DNA and (b) D-DNA tension probe surfaces. Scale bars = 10 μm

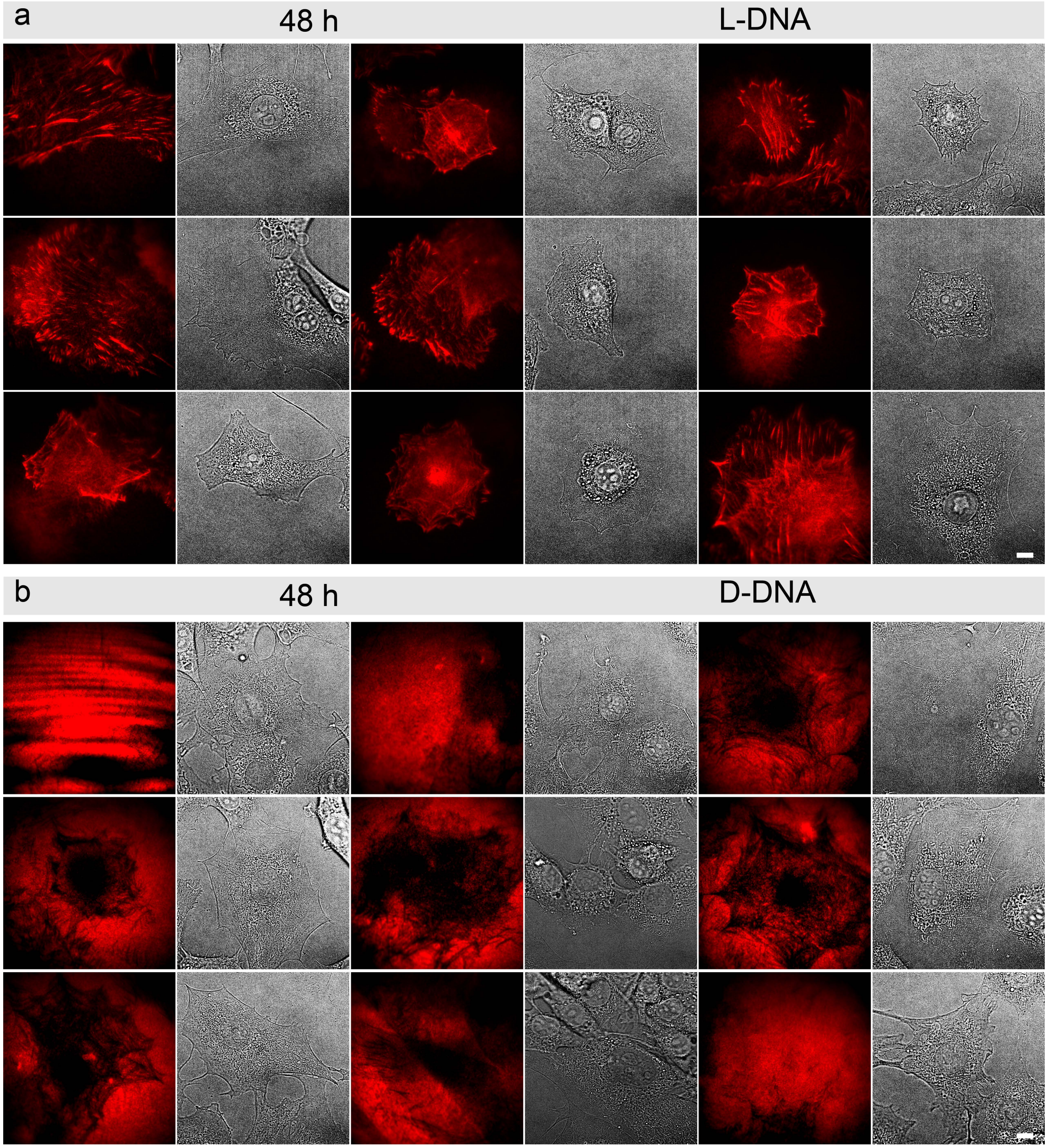

Figure S5. Representative Images of tension signals made by fibroblasts as they adhere, spread, crawl at 48 h time point (a-b) Brightfield images and fluorescence signals on (a) L-DNA and (b) D-DNA tension probe surfaces. Scale bars = 10 μm

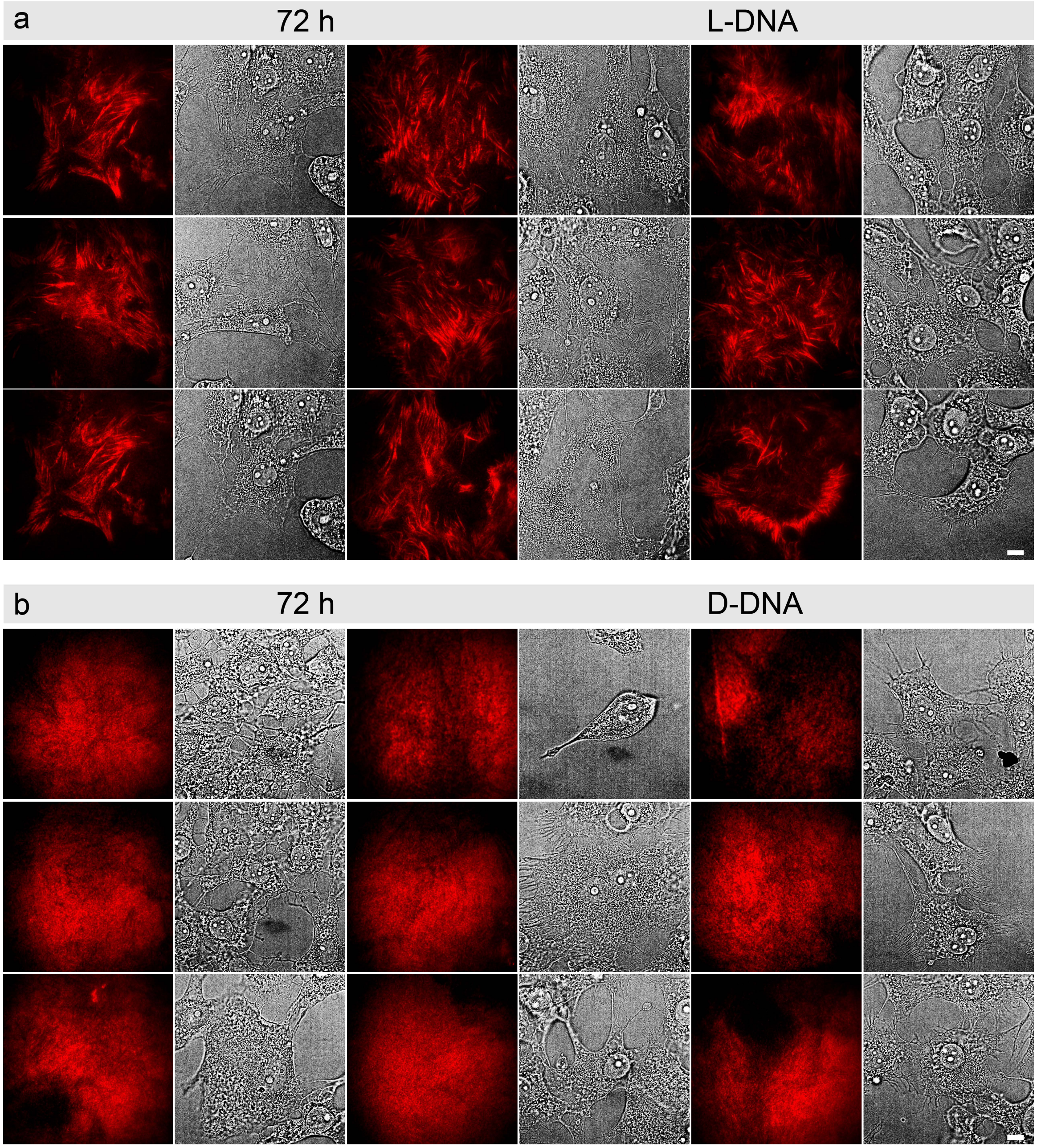

Figure S6. Representative Images of tension signals made by fibroblasts as they adhere, spread, crawl at 72 h time point (a-b) Brightfield images and fluorescence signals on (a) L-DNA and (b) D-DNA tension probe surfaces. Scale bars = 10 μm

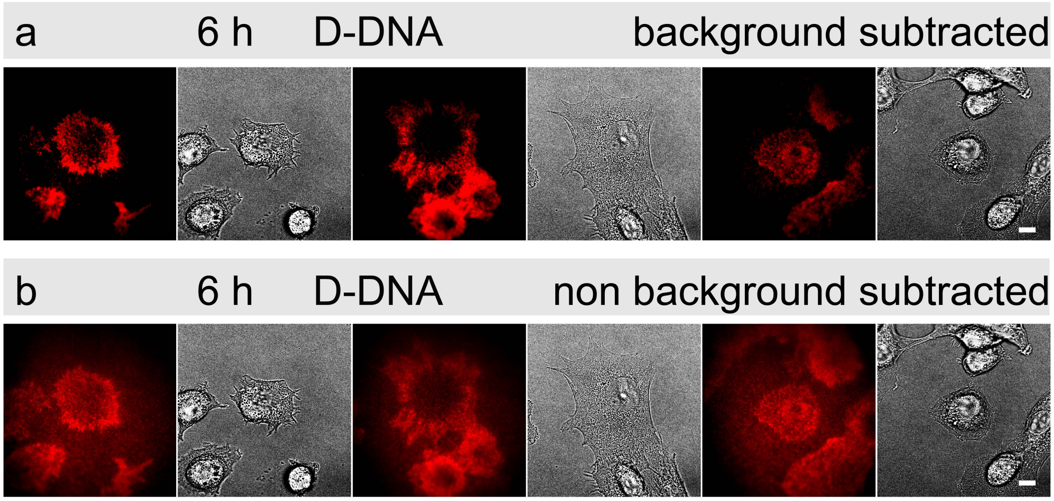

Figure S7. Representative Images of tension signals at 6 h time point on D-DNA surfaces. (a) Images with routinely used background subtraction (b) Images without any background subtraction. Scale bars = 10 µm

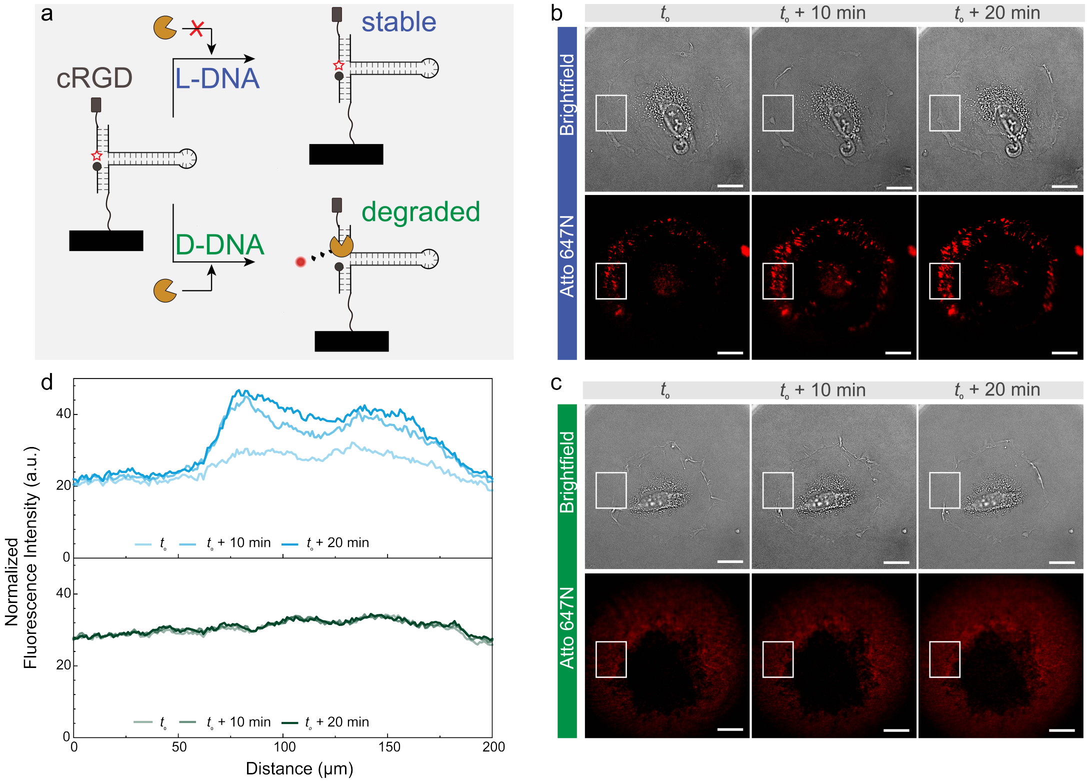

Figure S8. Repetition of experiment in Figure 3. Comparison of D-DNA and L-DNA reversible hairpin probes to study membrane ruffling in myoblasts (A-10). (a) Schematic depicting the geometry of hairpin tension probes and the stability in cell culture media. The used hairpin probes have 16 pN opening force. (b-c) Time lapse brightfield and fluorescence images on (b) L-DNA and (c) D-DNA hairpin tension probe surfaces 18 h after cell seeding. Consecutive images depict the ruffling of the myoblast cell membrane as illustrated by a cross-sectional region of interest (ROI). Scale bar = 20 µm. (d) Cross sectional horizontal analysis of the fluorescence intensity (normalized between [0,100]) at different time points within the ROI from b and c depicting ruffling of the cell membrane as the cell migrates forward.

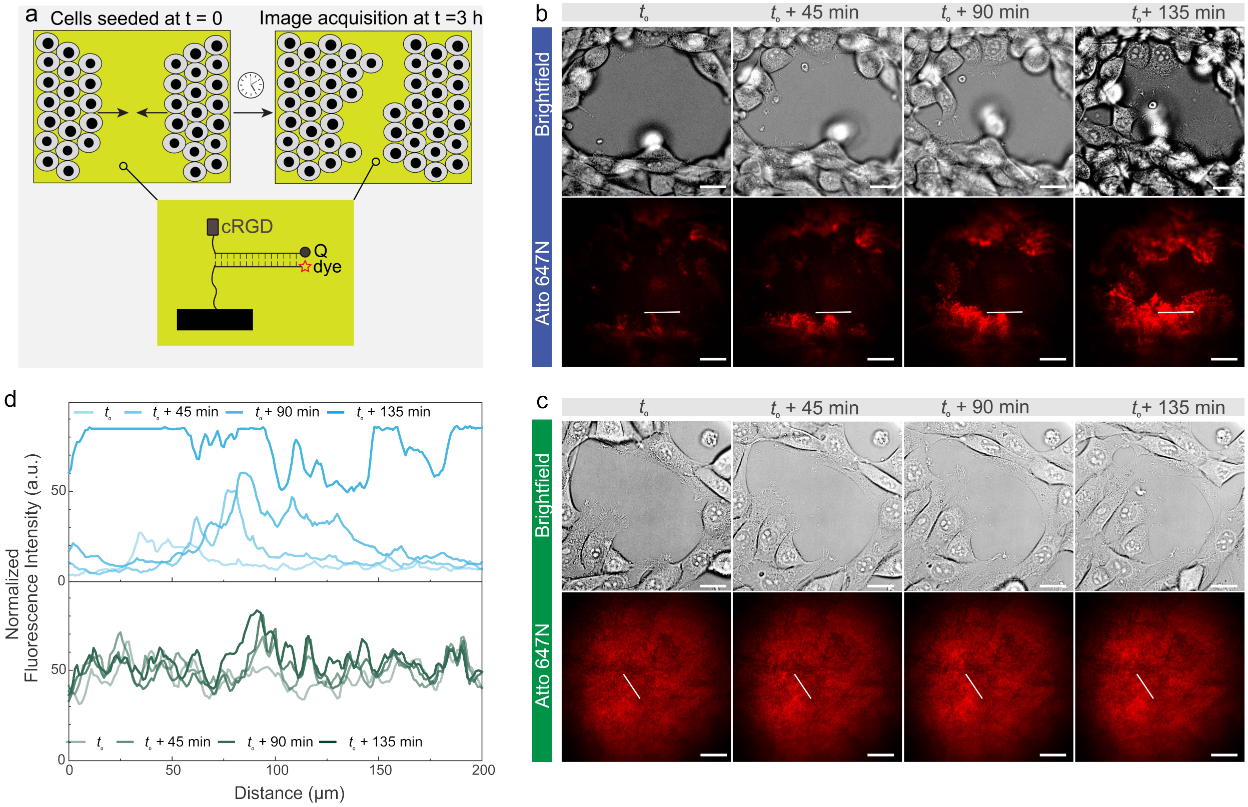

Figure S9. Repetition of experiment in Figure 4. Mapping forces during collective cell migration. (a) Schematic representation (b-c) Time lapse Brightfield and fluorescence images as fibroblasts migrate collectively on (b) L-DNA and (c) D-DNA tension probe-coated surfaces. Captured 3 h after seeding, and removal of a 500 µm barrier separating two fibroblast sheets. Scale bars = 20 µm. (d) Overlay of selected fluorescence intensity (normalized between [0,100]) cross sections from b and c. Note that dim edges and corners in c result from heterogeneous illumination normal to TIRF imaging.

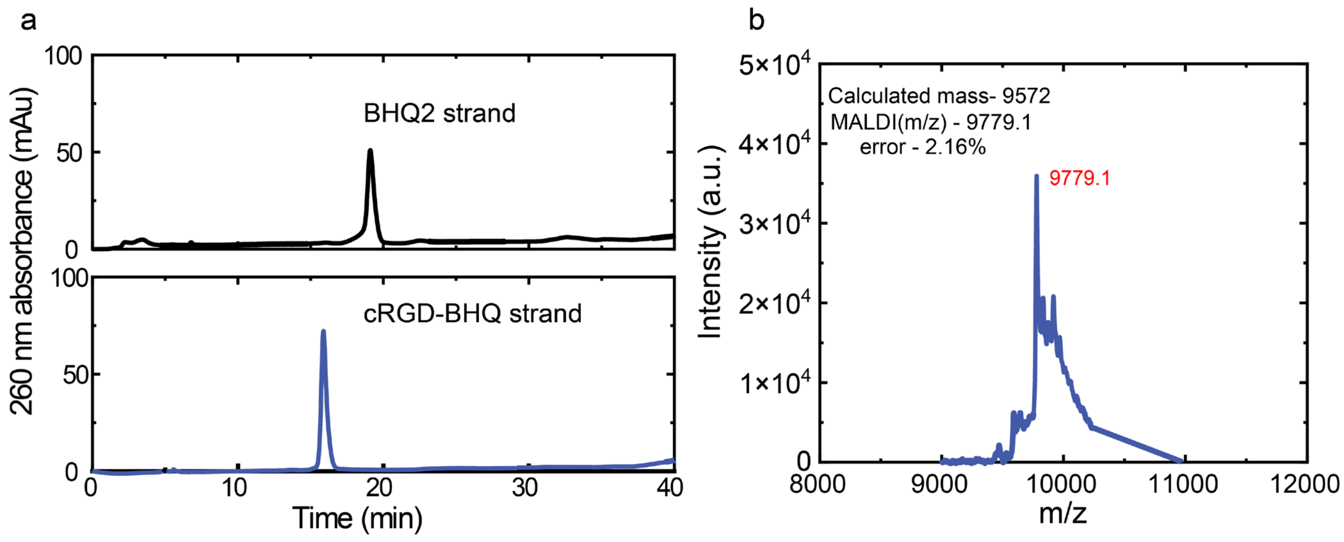

Figure S10. Characterization of DNA conjugated with cRGD. (a) HPLC graphs depicting different elution times of DNA-BHQ ssDNA and cRGD-DNA-BHQ ssDNA. (b) MALDI-ToF MS depicting the observed molecular weight of the cRGD-DNA-BHQ ssDNA product. Calculated for [M+9Na] + = 9779, Found [M+9Na] + = 9779.1.

6. Supplementary References

[1] M. R. Pawlak, A. T. Smiley, M. P. Ramirez, M. D. Kelly, G. A. Shamsan, S. M. Anderson, B. A. Smeester, D. A. Largaespada, D. J. Odde, W. R. Gordon, *Nat. Commun.* **2023**, *14*, 2468.

[2] Y. Zhang, C. Ge, C. Zhu, K. Salaita, *Nat. Commun.* **2014**, *5*, 5167.
